# Formulation of Chitosan-Coated Brigatinib Nanospanlastics: Optimization, Characterization, Stability Assessment and In-Vitro Cytotoxicity Activity against H-1975 Cell Lines

**DOI:** 10.3390/ph15030348

**Published:** 2022-03-13

**Authors:** Randa Mohammed Zaki, Munerah M. Alfadhel, Saad M. Alshahrani, Ahmed Alsaqr, Layla A. Al-Kharashi, Md Khalid Anwer

**Affiliations:** 1 Department of Pharmaceutics, College of Pharmacy, Prince Sattam Bin Abdulaziz University, P.O. Box 173, Al-Kharj 11942, Saudi Arabia; r.abdelrahman@psau.edu.sa (R.M.Z.); m.alfadhel@psau.edu.sa (M.M.A.); a.alsaqr@psau.edu.sa (A.A.); 2 Department of Pharmaceutics and Industrial Pharmacy, Faculty of Pharmacy, Beni-Suef University, Beni-Suef 62514, Egypt; 3 Department of Pharmacology and Toxicology, Faculty of Pharmacy, King Saud University, P.O. Box 2457, Riyadh 11451, Saudi Arabia; lalkharashi@ksu.edu.sa

**Keywords:** brigatinib, nanospanlastics, optimization, chitosan, sustained release, cytotoxicity

## Abstract

The purpose of the current study was to develop Brigatinib (BGT)-loaded nanospanlastics (BGT-loaded NSPs) (S1-S13) containing Span 60 with different edge activators (Tween 80 and Pluronic F127) and optimized based on the vesicle size, zeta potential (ZP), and percent entrapment efficiency (%EE) using Design-Expert^®^ software. The optimum formula was recommended with desirability of 0.819 and composed of Span-60:Tween 80 at a ratio of 4:1 and 10 min as a sonication time (S13). It showed predicted EE% (81.58%), vesicle size (386.55 nm), and ZP (−29.51 mv). The optimized nanospanlastics (S13) was further coated with chitosan and further evaluated for Differential Scanning Calorimetry (DSC), X-ray Diffraction (XRD), in vitro release, Transmission Electron Microscopy (TEM), stability and in-vitro cytotoxicity studies against H-1975 lung cancer cell lines. The DSC and XRD revealed complete encapsulation of the drug. TEM imagery revealed spherical nanovesicles with a smooth surface. Also, the coated formula showed high stability for three months in two different conditions. Moreover, it resulted in improved and sustained drug release than free BGT suspension and exhibited Higuchi kinetic release mechanism. The cytotoxic activity of BGT-loaded SPs (S13) was enhanced three times in comparison to free the BGT drug against the H-1975 cell lines. Overall, these results confirmed that BGT-loaded SPs could be a promising nanocarrier to improve the anticancer efficacy of BGT.

## 1. Introduction

Lung cancer is the second most common types of cancer in the United States and the main cause of cancer mortality. In 2020, an anticipated 247,270 new instances of lung cancer would be diagnosed, including 130,340 male cases and 116,930 female cases [1,2]. Anticancer drugs are considered successful when they exhibit maximum activity at target cancer cells, that can be achieved by a targeted drug delivery system [3]. Nanomaterials are an optimal choice as a targeted delivery system for the delivery of anti-cancer drugs by electively localizing them in tumor cells, lowering the risk of harm to healthy cells. This then reduces toxicity with increased efficacy [4].

Brigatinib (BGT) is a second-generation anaplastic lymphoma kinase (ALK) inhibitor that is used to treat a certain type of non-small cell lung cancer (NSCLC) by inhibiting an abnormal protein that causes cancer cells to multiply [5,6,7]. This slows or stops the spread of cancer cells [8]. It was approved by the Food and Drug Administration (FDA) in April 2017 and used to treat patients with advanced stages of metastatic ALK-positive NSCLC who are resistant to certain other ALK inhibitors including crizotinib, ceritinib, and alectinib [9]. BGT is available in a tablet form (30, 90, and 180 mg) with the best dose being 180 mg once a day for NSCLC. This drug has received the attention of researchers to formulate it in different forms in order to improve its action at the targeted cancer cells.

Ansari et al., 2020 [10] developed a self-nanoemulsifying drug delivery system (SNEDDS) of BGT, to enhance permeability of BGT to the targeted site. They found that cytotoxic activity of BGT-SNEDDS against A549 human lung cancer cell lines during WST 1 assay were significantly improved, as compared to pure BGT. Camidge and co-workers found that patients who received BGT had a substantially longer progression-free survival than those who received crizotinib in patients with ALK-positive NSCLC [11]. Moreover, BGT was reported to have clinically relevant effectiveness in Japanese patients with ALK+ NSCLC resistant to alectinib (with or without previous use of crizotinib) [12]. Also, BGT-loaded ethyl cellulose nanosponges and solid lipid nanoparticles were formulated for prolonged drug release to extend anti-cancer activity and found that BGT-loaded nanosponges and solid lipid nanoparticles dramatically reduced the cell viability of A549 human lung cancer cell lines [13,14].

Nanovesicles (liposomes and niosomes) can be used for the treatment of cancer with excellent results. It can also improve the stability of encapsulated drugs [15,16]. These conventional carriers, on the other hand, are rigid and lack deformability while passing through biological membranes. As a result, current research has focused on enhancing the deformability of these traditional nanovesicles to improve their permeability across biological membranes [17]. Nanospanlastics (NSPs) are flexible nanovesicles that are non-immunogenic, biodegradable, and harmless. They’re also more chemically stable than conventional liposomes [16]. For these reasons, several studies are focusing on the use of NSPs formulations as a promising delivery system in preference to the conventional nanovesicles.

NSPs are a highly elastic surfactant-based deformable nanocarrier system that were developed by Kakkar and Kaur [18]. Non-ionic surfactant (Span-60 and Span-80) and an edge activator (EA) are the key components of NSPs. The role of EA is in destabilizing the nanocarrier vesicular membranes, by squeezing through the narrow pores of the biological membranes without rupture, enhancing their flexibility and permeability across the biological membranes [19,20]. To the best knowledge of the authors, NSPs formulations of BGT have not yet been investigated in literature, in spite of several favorable characteristics of BGT including poor water-solubility.

Nanovesicles have a tendency to aggregate/merge leading to drug leakage during storage. Furthermore, there is a risk of fast blood clearance following intravenous injection when employing such drug carrier systems. Surface coating the vesicles with polymers leads to an increase in their stability, lengthening of their life in the blood stream, and offers sustained release of the contained medicine. The polymer chitosan was selected to coat the NSPs in order to target medications to maximize their absorption [21]. Chitosan, a natural polysaccharide derived from marine crustaceans, mollusks, insects, and fungi, is of great interest, particularly in drug delivery and biomedical application [22,23]. It may be processed into a variety of forms for various uses, including solutions, gels, mixes, sponges, tablets, membranes, and paste.

The current study includes formulation and evaluation of BGT-loaded SPs containing Span with different EA (Tween 80 and Pluronic F127) to improve the solubility, permeability of BGT. Optimization of the developed formulation was performed by Design Expert software to study the independent variables, namely, sonication time, type of EA, and Span-60:EA ratio on the dependent variables, entrapment efficiency, vesicles size, and zeta potential. Thereafter, the optimized NSPs were further coated and evaluated for DSC, XRD, in vitro release, TEM, stability and in vitro cytotoxicity studies against H-1975 lung cancer cell lines.

## 2. Results and Discussion

### 2.1. Analysis of I Optimal Design for Optimization of BGT Loaded NSPs

Design Expert Version 12.0.3.0 was used to study the effect of the independent variables namely, sonication time (X1), type of EA (X2), and Span-60:EA ratio (X3) on the dependent variables, entrapment efficiency (EE%) (Y1), vesicles size (Y2), and zeta potential (Y3) of BGT-loaded SPs according to I optimal design (Table 1).

Regression equations exhibited the effect of independent variables on the experimentally studied dependent responses by comparing the factor coefficients. In this model, adequate precision values for responses Y1, Y2, and Y3 were found greater than 4 as 51.9945, 95.8437, and 24.4528, respectively, hence this model could be used successfully for experimental design [24]. The data of responses Y1, Y2 and Y3 exhibited excellent linearity with their R^2^ values as 0.9993, 0.9998 and 0.9951, respectively. Hence, the obtained equations were found to be statistically valid and an excellent fit to the obtained data [25]. The predicted R^2^ values measured the response value consistency gave knowledge on how good the model could fit with the new results that came from the same relationship that was modeled. The adjusted R^2^ value is the modified form of R^2^ value that examines how well the present model would fit to the observed results. Subsequently, the predicted and adjusted R^2^ should be close to each other. In the event that they are not, there might be a problem with either the model or data. The difference between the predicted and adjusted R2 of Y1, Y2 and Y3 values were found to be less than 0.2, suggesting reasonable agreement [26].

### 2.2. Evaluation of the Prepared BGT Loaded SPS

#### 2.2.1. Entrapment Efficiency (EE%)

The EE% values were found to be ranged from 45.8 ± 2.37 to 88.7 ± 1.31% (Table 2). So, BGT was successfully entrapped in the NSPs’ formulations, indicating that span 60 based nanovesicles can be used as a successful delivery system for BGT.

**Figure 1:**
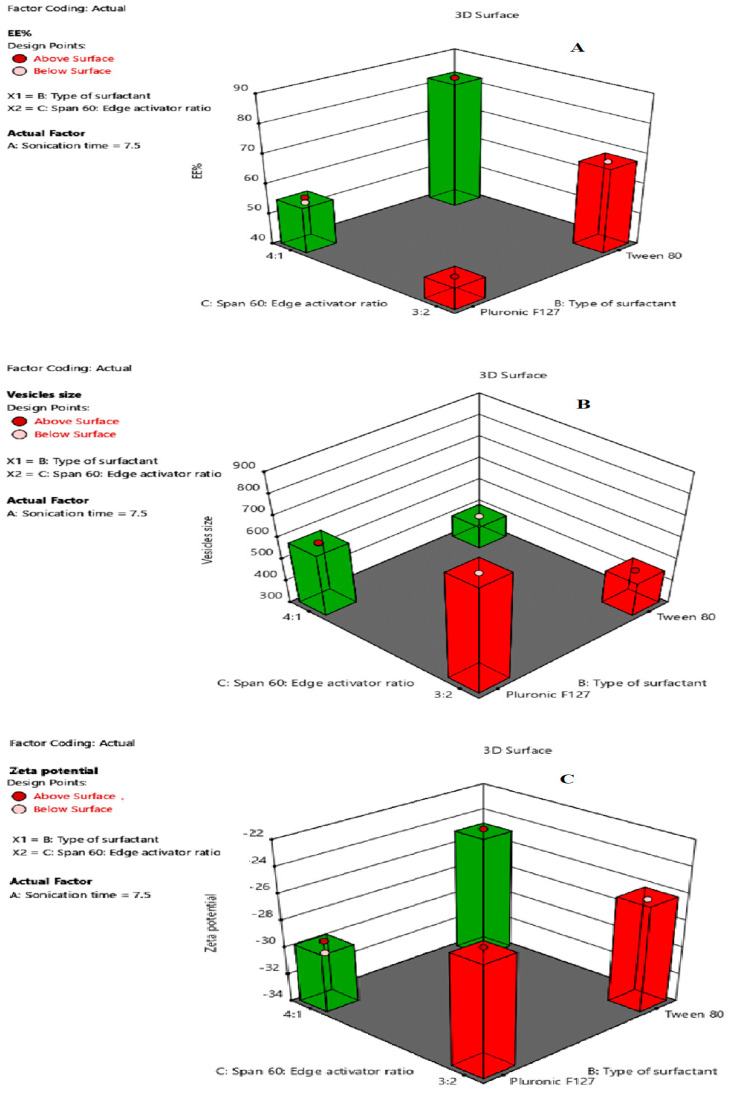
Response surface plots for the effects of type of surfactant (X1), Span-60:EA ratio (X2), and sonication time (X3) on: (**A**) EE%; (**B**) vesicles’ size; and (**C**) zeta potential, respectively.

The EE% values were substantially influenced by all the independent variables (*p* < 0.05). The effects of the type of surfactant (X1), Span-60:EA ratio (X2), and sonication time (X3) on EE% are represented in Figure 1A. ANOVA suggested a quadratic model with a F-value of 239.68 (*p* < 0.05) (Table 1), indicating a significant model. The following regression equation describes the effect of independent variable on %EE:

Y1 = +63.49 − 3.70A + 12.21B + 5.69C + 0.1250AB − 0.3000AC + 1.54BC + 2.13A2 + 0.2750ABC − 0.6591 A2B+ 0.7841A2C(1)

where A is the sonication time, B is the type of surfactant, and C is span 60:EA ratio. The positive and negative sign in the equation indicated the favorable and unfavorable nature of independent variables over the response [24]. It is clear from the regression Equation (1) that the sonication time had a negative impact on EE%, while the type of surfactant, and span 60:EA ratio had positive effects on EE%.

Regarding the effect of sonication time, it was noted that increasing the sonication time from 5 min (S1) to 10 min (S13) significantly decreased the EE% from 88.7 to 81.5%, possibly attributed to decreasing the vesicular size of NSPs or the escape of BGT to the external aqueous medium during disruption and re-aggregation of nanovesicles and retention there, rather than encapsulation in the nanovesicles [27]. These results were in agreement with those of Elsherif et al. [28], who studied the transungual delivery of Terbinafine Hydrochloride-loaded NSPs.

With respect to the type of surfactant, Tween^®^ 80-based NSPs (S1, 88.7%) showed higher EE% than Pluronic F127-based NSPs (S5, 63.5%). This could be explained on the bases of the hydrophilic–lipophilic balance (HLB) of EA which were 15 and 22 for Tween^®^ 80 and Pluronic F127, respectively [29,30]. So, Tween^®^ 80 was more hydrophobic than Pluronic F127, therefore, Tween^®^ 80-based NSPs were more rigid by decreasing the amphiphilic property of the vesicles’ membrane, resulting in higher EE% [31]. These results are in agreement with those of Abdelbari et al. [32] who reported higher EE% with Tween^®^ 80-based splanlastics than Pluronic F127-based NSPs for the ocular delivery of clotrimazole.

Additionally, changing the ratio of span 60:EA from 3:2 to 4:1 significantly enhanced the EE%, possibly due to the higher content of span 60 that caused a reduction in the fluidization of the NSPs membrane and, consequently, decreased the leakage of BGT, thus, enhancing the EE%. These results are in agreement with those of Badria and Mazyed [33] who investigated the effect of span 60:EA ratio on EE% of (3-Acetyl-11-Keto -β-Boswellic Acid)-loaded NSPs.

#### 2.2.2. Vesicles’ Size and Size Distribution PDI

Vesicles’ size is a very important criteria for enhancing the cell delivery of BGT. All BGT-loaded SPs showed vesicles’ size in the range of 388 ± 8.93 to 832.2 ± 23.34 nm (Table 2), indicating a nanosize range. The vesicles’ size values were substantially influenced by all independent variables (*p* < 0.05). The effects of the type of surfactant (X1), Span-60:EA ratio (X2), and sonication time (X3) on vesicles’ size are represented in Figure 1B. The ANOVA of the model indicated a significantly fitted quadratic model with F-value 1065.42, as presented in Table 1.

The regression equation for the effect of independent variable on vesicles size is given below:

Y2 = +562.35 − 28.86A − 130.95B − 64.90C − 3.61AB − 1.11AC + 38.99BC + 12.04A2 − 5.26ABC + 3.69A2B + 4.19A2C(2)

where A is the sonication time, B is the type of surfactant, and C is span 60:EA ratio.

It is worth noting from the regression Equation (2) that all independent variables (A, B, and C) had negative effects on vesicles’ size values. Regarding the effect of sonication time, it was found that increasing the sonication time from 5 min to 10 min significantly decreased the vesicles’ size from 465.7 (S1) to 388 nm (S13). This finding is in accordance with Elsherif et al. [28], who reported a decrease in the vesicles’ size of Terbinafine Hydrochloride-loaded NSPs upon increasing the sonication time. With respect to the type of surfactant, formulations prepared with Pluronic F127 (S5, 621.6 nm) showed larger vesicles’ size than those prepared with Tween^®^ 80 (S1, 465.7 nm), this could be attributed to the higher hydrophilicity of Pluronic F127 (HLB > 20) than Tween^®^ 80 (HLB = 15), leading to greater water uptake by the vesicle membranes and thus increasing in the vesicles’ size. Using EA of a lower hydrophilicity (lower HLB) caused a decrease in the surface energy and hence formation of smaller size nanovesicles [34].

Moreover, the Span-60:EA ratio 3:2 showed larger vesicles’ size than those of 4:1 ratio and this may be due to the higher EA concentration that led to the larger vesicles’ size. Both Tween^®^ 80 and Pluronic F127 are hydrophilic nonionic surfactants [35] that impart flexibility to the bilayer membranes of NSPs [36] and, thus, increase the elasticity of the vesicles and water uptake so leading to an increase in the vesicles’ size. PDI is an indicator of the vesicles’ size distribution and its value ranges from 0.0 (for completely uniform vesicles’ size distribution) to 1.0 (for highly polydispersed vesicles). The PDI values were found to be in the range 0.235 ± 0.10 to 0.648 ± 0.07, confirming low variation in the vesicles’ sizes (Table 2).

#### 2.2.3. Zeta Potential

Zeta potential is a measure for vesicles’ attraction or repulsion. Therefore, it is used to predict the nanovesicles’ stability. The higher the zeta potential values, the higher the stability. Formulations with zeta potential values greater than +30 or less than −30 are highly stable systems [37]. All BGT-loaded SPs showed that the zeta potential values ranged from −22.4 ± 1.48 to −33.2 ± 1.73 mv (Table 2), indicating a low tendency for NSPs aggregation and, consequently, high stable nanoformulations.

The zeta potential values were substantially influenced by all independent variables (*p* < 0.05). The effects of the type of surfactant (X1), Span-60: EA ratio (X2), and sonication time (X3) on zeta potential are shown in Figure 1C. ANOVA suggested a quadratic model with F-value 67.62 (*p* < 0.05), indicating a significant model (Table 1).

The following regression equation describes the effect of independent variable on zeta potential:

Y3 = −26.53 − 3.89A + 1.13B − 0.6358C − 0.2125AB + 0.1375AC + 1.27BC − 0.5300A2 + 0.9125ABC − 1.17A2B(3)

where A is the sonication time, B is the type of surfactant, and C is span 60:EA ratio. It is clear from the regression equation that the zeta potential values are significantly affected by all three independent factors at (*p* ˂ 0.05).

It was clear that the sonication time had a negative impact on the zeta potential values although it showed an interactive effect with both the surfactant type and span 60:EA ratio. As shown in Equation (3), sonication time and the type of surfactant collectively showed a negative impact on the zeta potential which may be attributed to the main effect of sonication time. Similarly, although sonication time and span 60:EA ratio showed a negative effect separately, they collectively showed a positive effect on the zeta potential values.

**Figure 2:**
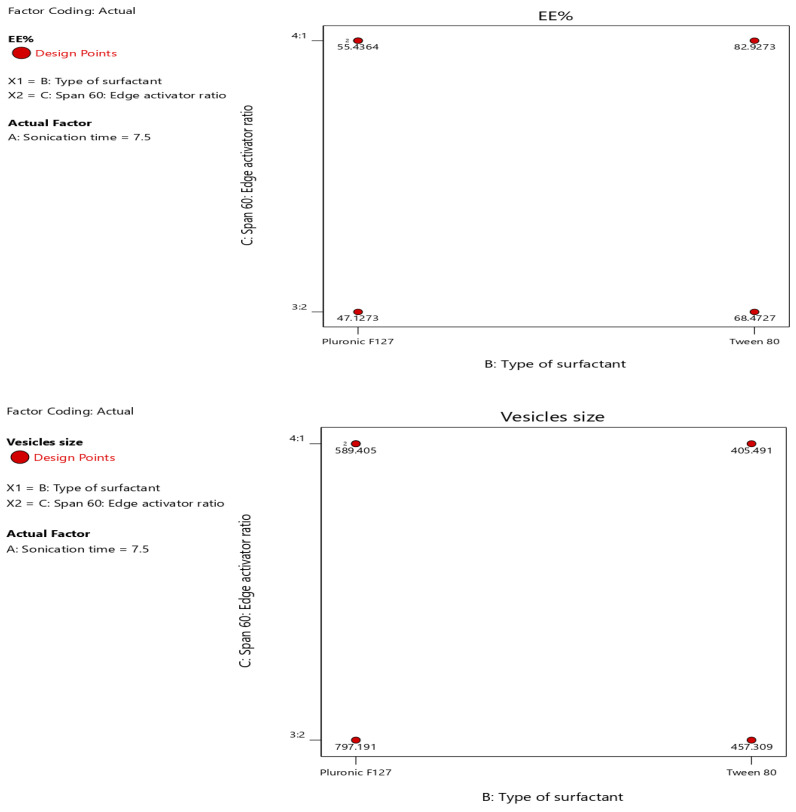

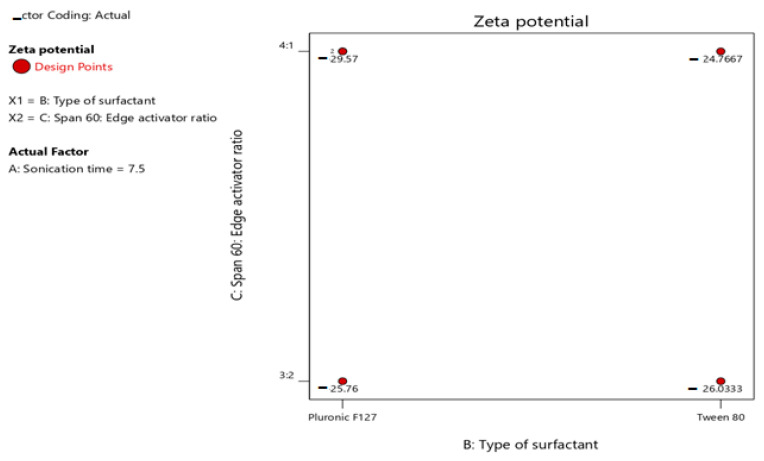
Contour plot of different responses.

Moreover, the type of surfactant showed a positive impact and the span 60:EA ratio showed a negative impact on the zeta potential values but collectively showed a positive impact mainly due to the effect of surfactant type. Moreover, the effect of different independent factors (X1, X2, X3) on different dependent variables (Y1, Y2, Y3) was represented as a contour plot (Figure 2).

### 2.3. Selection of the Optimized BGT Loaded SPs

**Figure 3:**
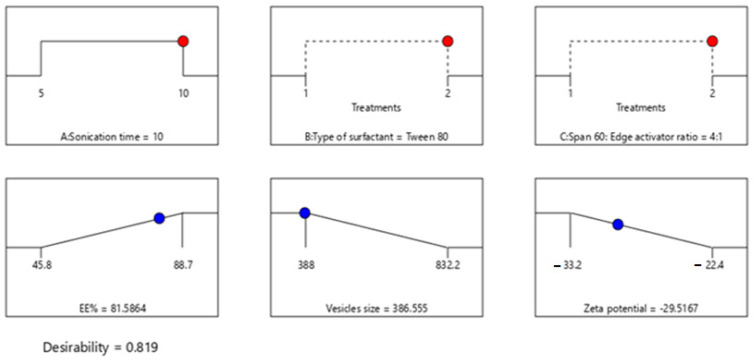
The composition of the optimized formula and its expected responses.

**Figure 4:**
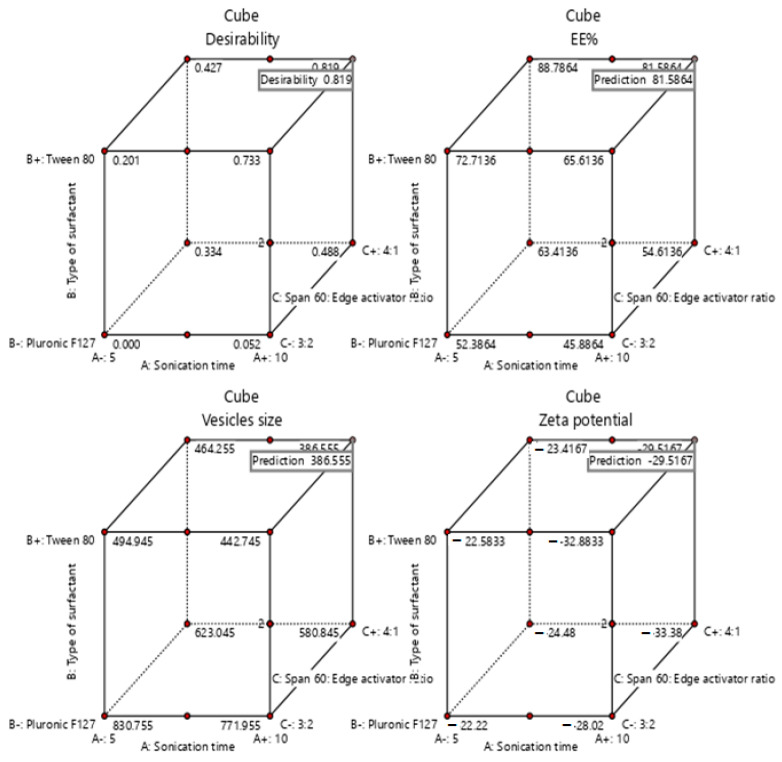
Cube graph for the predicted responses of the optimized formula and desirability.

Design-Expert^®^ software was used for optimization by choosing the formula of high desirability index. The principle of the software to select the optimized formula is based on maximizing (Y1) and (Y3), while minimizing (Y2). The optimum formula composed of Span-60:Tween^®^ 80 at a ratio of 4:1 and 10 min as a sonication time (formula S13), with a desirability of 0.819 (Figure 3). Additionally, the % relative error was calculated and found to be 3.04, 1.64, 3.02 for Y1, Y2, and Y3, respectively. These results were less than 5, indicating fitness of the model. The data were represented as a cube graph for the predicted responses and desirability of the optimized formula (Figure 4).

### 2.4. Evaluation of the Optimized Coated Formula

#### 2.4.1. Vesicle Size, %EE, and Zeta Potential

The optimum coated formula showed an increase in the EE% (86.55%) compared to the uncoated optimum formula (81.58%), possibly due to the change in the surface properties of the NSPs that prevents the leakage of the drug after chitosan coating, as reported by Alshraim et al. [38]. Additionally, the vesicles’ size increased from 386.55 to 395.4 nm, indicating the binding of chitosan to the surface of the NSPs [39]. Moreover, the coated formula showed a shift for zeta potential value from negative to positive, confirming the presence of the chitosan coating on the external surface of NSPs. This result is in agreement with Cuomo et al. [40].

#### 2.4.2. Differential Scanning Calorimetry (DSC)

**Figure 5:**
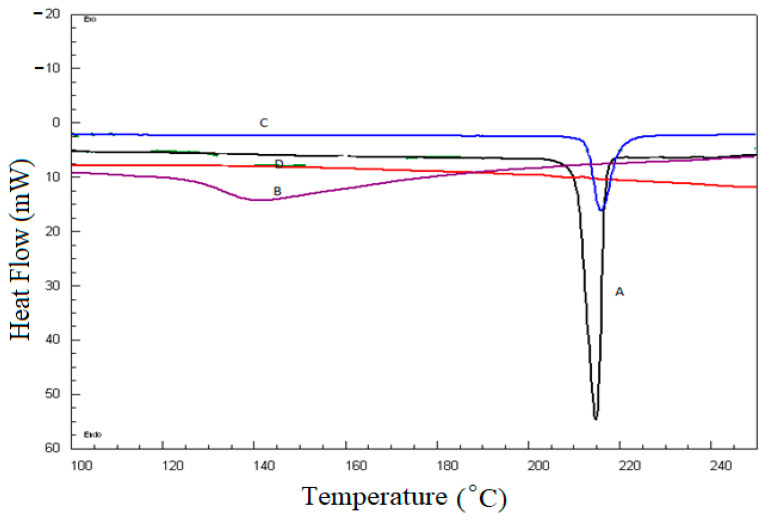
Comparative DSC thermograms; A. pure BGT; B. Span-60, Tween^®^ 80, and chitosan physical mixture; C. Span-60, Tween^®^ 80, Chitosan, and BGT physical mixture; D. the optimized formula.

DSC thermal analysis was performed to identify the possible interaction between pure BGT and excipients. A comparative DSC spectrum is presented in Figure 5. The crystallinity of the pure drug BGT was confirmed by a sharp endotherm at 215 °C (Figure 5 (A)), which was an agreement with previously reported studies [14]. The DSC thermogram of Span-60, Tween^®^ 80, and the chitosan physical mixture (Figure 5 (B)) showed an endothermic peak at 142 °C due to the melting transition of chitosan [41]. The DSC thermogram of Span-60, Tween^®^ 80, Chitosan, and the BGT physical mixture showed a sharp endothermic peak of BGT with reduced intensity at 215 °C (Figure 5 (C)), indicating no chemical interaction between the BGT’s and NSPs’ ingredients. Additionally, the optimized formulation (Figure 5 (D)) showed complete disappearance of the endothermic peak of BGT, confirming complete drug encapsulation within NSPs vesicles.

#### 2.4.3. X-ray Diffraction (XRD) Analysis

**Figure 6:**
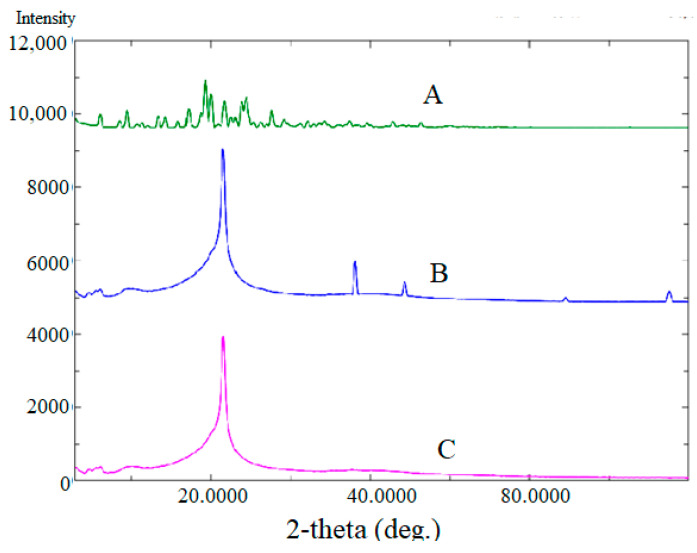
Comparative XRD of spectra of pure BGT (A); Span-60, Tween^®^ 80, and chitosan physical mixture (B); and chitosan-coated BGT-loaded NSPs (C).

XRD spectra of pure BGT and chitosan-coated BGT-loaded NSPs were presented in Figure 6. The XRD pattern of pure BGT drug (Figure 6 (A)) evidenced with various intense peaks at 2θ values of 6.4°; 8.1°; 9.9°; 10.8°; 11.3°; 13.7°; 14.3°; 15.9°; 17.2°; 18.9°; 19.4°; 21.2°; 22.6°; 23.4° and 28.2°, confirming the crystalline form of the drug [42]. The physical mixture of Span-60, Tween^®^ 80, and chitosan did not show any drug peak and one sharp peak of excipient could be seen (Figure 6 (B)), however complete disappearance of the drug peak was noted in the XRD spectrum of the optimized formula (Figure 6 (C)), only one intense peak near 22° could be seen, probably due to the coating of chitosan. The results indicated complete encapsulation of drug within NSPs’ vesicles.

#### 2.4.4. In Vitro Drug Release

**Figure 7:**
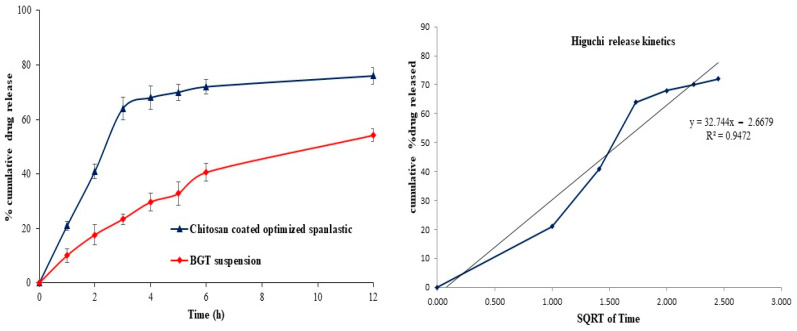
In-vitro release profile of chitosan-coated NSPs and pure BGT and Best fitted Higuchi release kinetic model.

The in vitro release profile of BGT from the chitosan-coated BGT-loaded NSPs compared to pure BGT suspension are presented in Figure 7. As shown in Figure 7, 64 ± 1.8% drug was released in the first three hours of the study followed by sustained release, this initial rapid release of drug from chitosan-coated BGT-loaded NSPs was due to the surface adsorbed drug on chitosan polymer. The chitosan-coated BGT-loaded NSPs exhibited enhancement in cumulative release of BGT (79.3 ± 2.4%) as compared to pure BGT suspension (54.2 ± 3.2%) in 12 h of the study. The release was found to be statistically difference (*p* < 0.01) from chitosan-coated BGT-loaded NSPs as compared to pure BGT suspension. The release data of chitosan-coated optimized NSPs were fitted with different kinetic models and the coefficient of correlation (R^2^) and slope are presented in Table 3. It was considered that the Higuchi model (Figure 7) acted as a best fit model, signifying sustained release of drug release from the chitosan polymer by diffusion control [43].

#### 2.4.5. Transmission Electron Microscopy (TEM)

**Figure 8:**
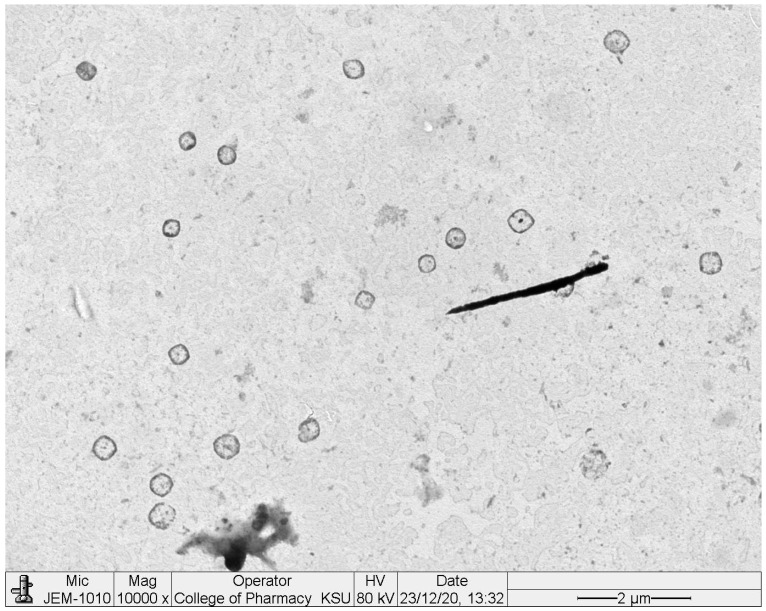
TEM images of chitosan-coated BGT-loaded NSPs.

TEM images of chitosan-coated BGT-loaded NSPs are shown in Figure 8. The images are shown as spherical in shape, having smooth surfaces with no aggregation of particles. The size of chitosan-coated BGT-loaded NSPs were observed as approximately same size, as measured by the DLS method.

#### 2.4.6. Stability Study

The stability of the optimized BGT-loaded NSPs was assessed in terms of EE%, vesicles’ size, zeta potential, and % drug release after storage at 4 °C and 37 °C for three months. After three months of storage at two different conditions, no significant changes in EE%, vesicles size, zeta potential, and % drug release were observed (Table 4). The stability data indicated that the optimized BGT-loaded NSPs was a stable formulation.

#### 2.4.7. Cytotoxicity Studies against H-1975 Cell Lines

**Figure 9:**
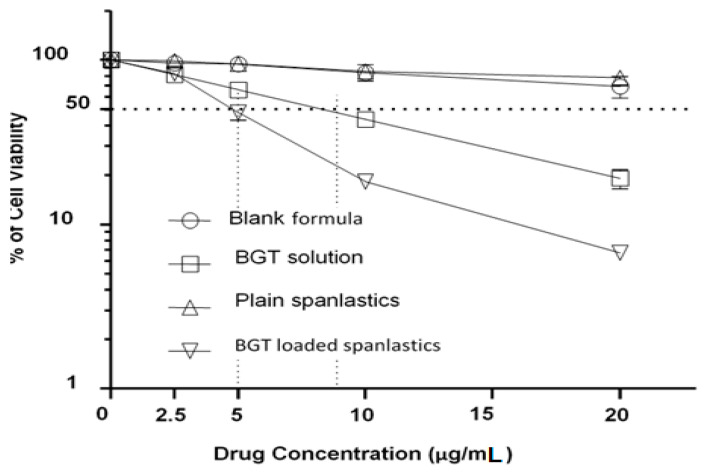
Cytotoxicity of BGT-loaded NSPs compared to plain NSPs, BGT solution, and blank formula on H-1975 NSCLC, as determined by a WST-1 assay. Cells were treated with varying concentrations of the drug as indicated for 48 h. Cell cytotoxicity was assessed using the WST1 assay and measured as % of survived cells relative to the non-treated control cells. Results obtained from three independent experiments. Error bars indicate means ± SD (*n* = 3).

Loading of BGT in chitosan-coated NSPs significantly improved the anticancer activity against H-1975 cell lines. The WST 1 assay exhibited a concentration-dependent reduction in percent cell viability by optimized BGT-loaded SPs (S13) in comparison to free BGT solution against H-1975 cell lines (Figure 9). The optimized BGT-loaded NSPs exhibited a significant reduction in cell viability (69.25, 38.51, 16.16 and 6.81% at 2.5, 5, 10 and 20 µg/mL) in comparison to free BGT (80.10, 69.45, 43.99 and 24.14% at 2.5, 5, 10 and 20 µg/mL) and blank NSPs (99.29, 89.98, 79.59 and 61.49% at 2.5, 5, 10 and 20 µg/mL), respectively. The anti-cancer effect of optimized BGT-loaded SPs (S13) was enhanced by 4.0-fold in comparison to free drug (BGT) at concentration 20 µg/mL against H-1975 cell lines. The reduction in cell viability by chitosan-coated BGT-loaded NSPs compared to BGT solution has been previously reported [13,14]. The enhanced cellular uptake of NSPs might be responsible for a decrease in cell viability [44].

## 3. Materials and Methods

### 3.1. Materials

Brigatinib was obtained from Mesochem Technology Ltd. (Beijing, China). High molecular weight Chitosan (310000–375000 Da), Tween^®^ 80, Span-60 and Pluronic F127 was purchased from Sigma Aldrich (St. Louis, MO, USA). NSCLC cells (H-1975 cells) were received from the American Type Culture Collection (ATCC, Manassas, VA, USA). Cells were incubated at 37 °C and cultured in RPMI 1640 medium (GIBCO^®^) containing 10% fetal bovine serum (FBS) and 1% antibiotic/antimycotic which were purchased from (GIBCO^®^, Invitrogen Corp, Carlsbad, CA, USA).

### 3.2. Experimental Design

I-optimal design was implemented using Design-Expert^®^ software (version 12.0.3.0, Stat-Ease, Inc., Minneapolis, MN, USA) to establish the optimum criteria for preparation of BGT-loaded NSPs. The independent variables were sonication time (X1), type of EA (X2), and Span-60:EA ratio (X3). The dependent variables were entrapment efficiency (EE%), vesicles’ size, and zeta potential, as represented in Table 1. The composition of different formulations recommended by the design expert along with its responses are shown in Table 1.

### 3.3. Development of BGT-Loaded NSPs

Different BGT-loaded NSPs were formulated by ethanol injection method with minor modification [45]. Two different surfactants (EA), namely, Pluronic F-127 and Tween^®^ 80, were used in the preparation as shown in Table 1. The amount of BGT was kept constant (30 mg), while Span-60 and the EA were used in different ratios. Briefly, accurately weighed Span-60 and BGT were dissolved in 5 mL of the organic phase consisting of chloroform:methanol (1:1, *v*/*v*), while the surfactant (EA) was dissolved in 10 mL aqueous phase separately. The organic phase was injected slowly into 10 mL of hot aqueous phase at a temperature of 50 °C, followed by continuous stirring at 1000 rpm for 1h. A white milky suspension of BGT-loaded NSPs was formed. Then the formed NSPs were sonicated for a time as specified in the design. Finally, all formulations were kept overnight in the freezer at 4 °C and kept in a tight closed container for further evaluation.

### 3.4. Entrapment Efficiency %

The free (unencapsulated) BGT was separated from different formulations by cooling centrifugation at 15,000 rpm and 4 °C for 1h. Later, the supernatant was isolated, filtered through a 0.45 μm filter, and suitably diluted to be evaluated for BGT content spectrophotometrically at 283 nm [10]. The experiment was repeated three times and the EE% was computed according to the following formula:

EE% = (intial added drug − free drug in supernatant)/(intial added drug) × 100)

### 3.5. Measurement of Vesicles Size, Size Distribution (PDI) and Zeta Potential (ZP)

The vesicles’ size of all NSPs formulations (S1-S13) were measured by Dynamic Light Scattering technique (DLS) using Zetasizer Nano ZS instrument (Malvern Instruments, Worcestershire, UK). The polydispersity index (PDI) was used to indicate the degree of distribution and uniformity of vesicles’ size. PDI values of less than <0.3 were considered monodispersed in size [46]. Zeta potential measurements give an indication of the magnitude of repulsion and attraction between vesicles as it measures the electric charges on the surface of nanovesicles. Therefore, zeta potential was used to predict the stability of nanovesicles. The zeta potential of all formulations was measured by a Malvern Zetasizer (Malvern Instruments, Worcestershire, UK) at 25 ± 1 °C. Freshly prepared samples were diluted (1:200), transferred into cuvette and analyzed for NSPs size, PDI and ZP.

### 3.6. Optimization of BGT Loaded SPs

Design-Expert^®^ software was applied to select the best formula by utilizing the desirability function [47]. The software selected the optimized formula based on maximizing EE% and zeta potential while minimizing the vesicles’ size. Additionally, to validate the optimized formula, the experimental values of EE%, vesicle size, and zeta potential were compared with the predicted values and the % relative error was calculated using the following formula [20].

% Relative error = (predicted value − experimental value)/predicted value) × 100

Furthermore, the optimized formula was prepared and coated with chitosan for further examinations.

### 3.7. Coating the Optimized Formula with Chitosan

The optimized formula was coated with 0.05 (%*w*/*v*) chitosan (high molecular weight). Briefly, 0.05% (*w*/*v*) chitosan was selected as a best concentration based on trials (unpublished data). Firstly, the accurate amount of chitosan was dissolved in 0.1% glacial acetic acid by the aid of magnetic stirrer. Then 10 mL of chitosan solution was added slowly to an equal volume of the optimized BGT-loaded NSPs, followed by magnetic stirring at 25 °C for 20 min. Finally, the coated formula was sonicated for 5 min for vesicles’ homogenization [48].

### 3.8. Evaluations for the Coated Optimized Formula

#### 3.8.1. Vesicles’ Size, % EE, and Zeta Potential

The coated optimized formula was evaluated for vesicles’ size, % EE and zeta potential, as previously described.

#### 3.8.2. Differential Scanning Calorimetry (DSC)

The thermal properties of pure BGT, additives, BGT-additives physical mixture, and the optimized BGT-loaded NSPs were examined by DSC (N-650; Scinco, Italy). Accurately weighed (5 mg) of each sample was pressed into a hermetically sealed aluminum pan, placed in DSC sample holder, and heated for a temperature that ranged from 50 °C to 250 °C at a heating rate of 10 °C/min [49]. The instrument was continuously purged with inert nitrogen gas with a flow rate 20 mL/min during experiment.

#### 3.8.3. X-ray Diffraction (XRD) Analysis

X-ray diffraction (XRD) analysis was performed to assess the crystalline state of pure BGT and optimized chitosan-coated BGT-loaded NSPs in Ultima IV Diffractometer (Rigaku Inc. Tokyo, Japan, at College of Pharmacy, King Saud University, Riyadh, KSA). The XRD spectra was scanned in the range of 0–80° (2θ) at a rate of 10°/min speed.

#### 3.8.4. In Vitro Drug Release

The in vitro release of BGT from the chitosan-coated BGT-loaded NSPs compared to pure BGT suspension was inspected employing the dialysis bags’ method. The chitosan-coated BGT-loaded NSPs and BGT suspension (each one equivalent to 5 mg BGT) were placed in the bags (Mol. Wt.:14 kDa). After that, the bags were suspended into beakers filled with 100 mL phosphate-buffered saline (pH 7.4) [13], kept at 37 ± 0.5 °C, with constant stirring at 100 rpm by a magnetic stirrer. At specific time intervals, samples of 2 mL were removed and replenished with an identical quantity of fresh medium to preserve the sink condition. Filtration for all samples were completed, followed by measuring BGT content spectrophotometrically at 283 nm [10]. All measurements were completed in triplicate.

Release data of chitosan-coated SPs were fitted with different kinetics’ models such as zero order, first order, Higuchi and Korsmeyer–Peppas models and were calculated by following equations [50]:

Zero order, Qt = Q0 + k0t

First order, logQt = logQ0 − k1t/2.303

Higuchi, Qt = kHt1/2

Korsmeyer–Peppas, Mt/M∞ = ktn

#### 3.8.5. Transmission Electron Microscopy (TEM)

The morphology and approximate vesicle size of optimized BGT-loaded NSPs were studied by TEM analysis (TEM; JEOL JEM-1010, Tokyo, Japan) [51]. The optimized BGT-loaded NSPs were diluted with Milli-Q water and vortexed for three minutes. A drop of suspended vesicles was put on parafilm, and the slide of the TEM grid was put on the drop and left for 10 min, The TEM grid with the slide was dried for 40 min using tissue paper, then scanned for vesicle imaging.

### 3.9. Stability Study

The optimized chitosan-coated SPs was kept in vials and stored at 4 °C and 37 °C in tightly closed glass vials for three months to estimate the presence of any aggregations, sedimentations or leakage during storage [52]. Samples were withdrawn and assessed for EE%, vesicles’ size, zeta potential, and in vitro release.

### 3.10. Cytotoxicity Studies against H-1975 Cell Lines

The in vitro cytotoxicity activity of chitosan-coated BGT-loaded NSPs, Plain NSPs (without drug), BGT solution, and blank formula (5%DMSO + 5%Methanol + 90%H_2_O) was analyzed using NSCLC cells (H-1975 cells) by WST-1 assay (WST-1; cat. No. ab155902; Abcam, Cambridge, UK). In brief, a total of 5000 cells/well were seeded into 96-well microtiter plates in a final volume of 100 µL appropriate culture medium and incubated overnight. All the samples with varying concentrations of 0, 2.5, 5, 10 and 20 μg/mL were added to each well and incubated for additional 48 h. After cell treatment, a 10 μL of WST1 reagent was added and incubated for 4 h at 37 °C. Blank control wells: 100 µL culture medium + 10 µL WST-1. The intensity of formazan dye was measured at 440 nm using an ELISA microplate reader (Thermo Fisher Scientific, Waltham, MA, USA). The % cytotoxicity was calculated by using the following formula;

% Cytotoxicity = (100 × (control-treated sample))/Control

### 3.11. Statistical Analysis

Results were expressed as the mean ± standard error of the mean (SEM). The Graph Pad prism software was used for statistical analysis.

## 4. Conclusions

In conclusion, the I-optimal Design-Expert^®^ assisted the BGT-loaded SPs to be successfully developed using formulation variables viz., non-ionic surfactant (Span-60) and EA (Twee 80, Pluronic F127), sonication time and Span-60:Edge activator ratio. The optimized BGT-loaded SPs (S13) showed a spherical image by TEM, and stable and improved anti-cancer activity against H-1975 lung cancer cell lines. The optimized BGT-loaded SPs (S13) were coated with chitosan polymer in order to sustain the release of BGT. A comparative drug release of optimized chitosan-coated BGT-loaded SPs showed improvement with a sustained release of BGT. Therefore, developed chitosan-coated brigatinib NSPs could be an alternate drug delivery system to overcome poor solubility of the drug.

## Figures and Tables

**Table 1:** Different variables utilized in I-optimal design for optimization of BGT-loaded NSPs.

Independent Variable		Levels					
		Low (−1)		High (+1)			
Sonication Time (X1)		5		10			
Type of EA (X2)		Pluronic F127		Tween 80			
Span-60: EA Ratio (X3)		3:2		4:1			
Dependent Variables	R^2^	Adjusted R^2^	Predicted R^2^	Constraints	*p* Value	F Value	Adequate Precision
Y1: % EE	0.9993	0.9959	0.9846	Maximize	0.0034	293.68	51.9945
Y2: vesicles size (nm)	0.9998	0.9989	0.9672	Minimize	0.0009	1065.42	95.8437
Y3: zeta potential (mV)	0.9951	0.9804	0.8818	Maximize	0.0026	67.62	24.4528

**Table 2:** I-optimal Design of BGT-loaded NSPs with their responses.

Formula Code	Sonication Time (min.)	Type of EA	Span-60:EA Ratio	EE (%)	Vesicles Size (nm)	Zeta Potential (mV)	PDIa
S1	5	Tween^®^ 80	4:1	88.7 ± 1.31	465.7 ± 11.45	−23.5 ± 1.03	0.441 ± 0.12
S2	10	Pluronic F127	3:2	45.8 ± 2.37	773.4 ± 15.46	−28.2 ± 0.74	0.627 ± 0.02
S3	5	Tween^®^ 80	3:2	72.8 ± 1.76	493.5 ± 14.32	−22.5 ± 2.31	0.573 ± 0.06
S4	7.5	Tween^®^ 80	3:2	68.3 ± 3.57	460.2 ± 8.56	−26.2 ± 1.76	0.351 ± 0.14
S5	5	Pluronic F127	4:1	63.5 ± 1.89	621.6 ± 22.47	−24.3 ± 1.45	0.432 ± 0.15
S6	7.5	Pluronic F127	3:2	47.3 ± 2.67	794.3 ± 13.45	−25.4 ± 0.83	0.631 ± 0.05
S7	5	Pluronic F127	3:2	52.3 ± 2.58	832.2 ± 23.34	−22.4 ± 1.48	0.507 ± 0.12
S8	7.5	Pluronic F127	4:1	56.2 ± 2.54	592.4 ± 21.76	−29.3 ± 2.43	0.235 ± 0.10
S9	7.5	Tween^®^ 80	4:1	83.1 ± 1.45	402.6 ± 16.54	−24.6 ± 1.98	0.436 ± 0.09
S10	7.5	Pluronic F127	4:1	54.5 ± 1.96	589.3 ± 18.43	−30.2 ± 2.45	0.602 ± 0.11
S11	10	Pluronic F127	4:1	54.7 ± 2.03	579.4 ± 25.43	−33.2 ± 1.73	0.648 ± 0.07
S12	10	Tween^®^ 80	3:2	65.7 ± 1.94	441.3 ± 17.65	−32.8 ± 2.45	0.553 ± 0.1
S13	10	Tween^®^ 80	4:1	81.5 ± 2.57	388 ± 8.93	−29.6 ± 1.85	0.474 ± 0.2

**Table 3:** Drug release kinetic models for optimized chitosan-coated NSPs.

Model Name	Chitosan-Coated NSPs	
	R^2^	Slope
Zero order	0.8655	17.179
First order	0.9174	0.0988
Higuchi	0.9472	32.744

**Table 4:** Values of EE%, vesicles’ size, zeta potential, and % drug release of the optimized coated BGT-loaded NSPs, initially and after storage for 3 months at 4 °C and 37 °C.

Parameter	Initial Values	After Storage at 4 °C	After Storage at 37 °C
EE%	86.5 ± 2.35	85.2 ± 1.56	83.8 ± 1.22
Vesicles’ size	395.4 ± 10.43	401.2 ± 6.45	405.3 ± 8.65
Zeta potential	33.2 ± 2.23	32.8 ± 1.24	31.7 ± 2.01
% Drug release	80.38 ± 3.24	78.5 ± 2.37	75.6 ± 3.57

## Data Availability

The data is contained in the manuscript.
